# Impact of work–life interference on burnout and job discontent: A one-year follow-up study of physicians in Sweden

**DOI:** 10.5271/sjweh.4181

**Published:** 2024-10-01

**Authors:** Britta E Gynning, Filip Christiansen, Ulrik Lidwall, Emma Brulin

**Affiliations:** 1 Unit of occupational medicine, Institute of Environmental Medicine, Karolinska Institutet, Stockholm, Sweden.; 2 Division of Insurance Medicine, Department of Clinical Neuroscience, Karolinska Institutet, Stockholm, Sweden; Department for Analysis, Swedish Social Insurance Agency, Stockholm, Sweden.

**Keywords:** healthcare worker, longitudinal, mental health, psychosocial work environment, well-being

## Abstract

**Objectives:**

In recent years, increased physician workload has led to higher levels of interference between work and private life with increasing stress and job discontent. The objective of this paper was to study if the experience of work–life interference (WLI) is associated with a high risk of burnout and discontent with work (turnover intention and job dissatisfaction) the following year among physicians in Sweden.

**Methods:**

The study applied data for 2021 and 2022 from the Longitudinal Occupational Health survey for Health Care professionals in Sweden study. The data comprised a representative sample of physicians (N=1575) working in Sweden. Descriptive analyses included frequencies and estimates of prevalence with Chi-square and McNemar tests. Analyses of association were assessed through logistic regression reporting odds ratios (OR) and 95% confidence intervals (CI) adjusting for demographics and work-related factors.

**Results:**

Higher levels of WLI in 2021 were associated with 1.53 (95% CI 1.05–2.25) times higher odds of reporting a high risk of burnout, 2.06 (95% CI 1.68–2.54) times higher odds of reporting job dissatisfaction, and 1.72 (95% CI 1.47–2.00) times higher odds of reporting turnover intention in 2022.

**Conclusions:**

Experiencing WLI negatively affects mental well-being and work satisfaction among physicians in Sweden. This could ultimately impact the quality of care and necessitates further research to clarify the role of WLI among healthcare workers in Sweden.

Physician burnout – a work-related state of exhaustion characterized by extreme tiredness, reduced ability to regulate cognitive and emotional processes, and mental distancing (1) – and discontent with work (ie, job dissatisfaction and turnover intention) constitute a major challenge for healthcare services and quality of care (2–6). To retain physicians at their jobs, research must identify work factors that drive and maintain ill health and discontent with work (7, 8).

In a recently published Swedish report, work–life interference (WLI), iei.e., incompatible role pressures stemming from the work domain to the private life domain (9, 10), was identified as a common work-related issue for physicians in Sweden (11). This is confirmed in international research, which shows that for physicians in Norway, Germany, and the United States, WLI has been and remains high (4, 6, 12–14). Thus, an important next step is to investigate whether WLI is associated with a subsequent high-risk of burnout and discontent with work among physicians in Sweden.

Indeed, evidence shows that WLI is related to high levels of strain and stress (13, 15, 16), burnout (4, 17), and, over time, an increase in sickness absence (18). Moreover, Lidwall et al (19, 20) have accentuated the increased risk of mental disorders in higher-status occupations when exposed to WLI. Fub et al (4) have observed similar results, which investigated variations in the impact of WLI between German physicians and the general German population. International studies also indicate an association between WLI and discontent with work (4, 17, 21).

Much of the current evidence on the association between WLI and ill-health and discontent with work, respectively, springs from cross-sectional studies (17). Meanwhile, many cross-sectional associations may disappear or change over time and more longitudinal studies are therefore needed to ascertain the direction and associations of WLI (17). Also, although physicians seem to be especially exposed to WLI, few studies have linked their WLI to burnout and discontent with work (17). Thus, this study aims to determine if WLI is associated with a high burnout risk and discontent with work, iei.e., job dissatisfaction and turnover intention, the following year among physicians in Sweden.

## Methods

This study applies data from the Longitudinal Occupational Health survey for HealthCare professionals in Sweden (LOHHCS). Baseline data was collected in 2021, including a sample of 6699 randomly selected physicians from the Swedish Occupational Register, based on 12 strata (the six care administrative regions of Sweden and primary versus hospital care facilities). The baseline response rate was 41.2% (22). The LOHHCS cohort is an open cohort, meaning that those who exited the labor market (iei.e., retired, died, or moved out of the country; N=697) were excluded from follow-up. The follow-up survey was distributed from March to June 2022. A total of 6002 physicians received the survey at both time points, with 1575 physicians (26.2%) responding at both measurement points.

Statistics Sweden, the Swedish Government agency administrating and producing official statistics, was responsible for sampling and collecting data. At each data collection, they analyzed missing data by comparing the responses to the sample and the population using the Swedish Total Population Register including individuals’ legal sex, age, birth country and civil registration conditions. We analyzed missing data by comparing those who received the survey in both 2021 and 2022 to those who only responded in 2021. The analysis showed that 57% of women and 59% of men answered both times. The prevalence of sickness absence was 10.0% among those only responding in 2021 and 9.3% in the analytical sample. The mean value of burnout in 2021 was 1.82 [standard deviation (SD) 0.63] for those who only responded in 2021 and 1.80 (SD 0.58) for our analytical sample. For WLI in 2021, the mean values and standard deviation were the same for both groups (2.96, SD 1.01).

### Study measures

*Exposure.* Baseline WLI was assessed using a subset of the scale proposed by Fisher and colleagues (10). WLI was measured using 5 items (supplementary material, www.sjweh.fi/article/4181, table S1) measured on a five-point Likert scale ranging from 1–5: “not at all” to “almost all the time”. The five items were compiled into a grand mean index (α:0.931), with a higher score indicating more WLI.

*Outcomes.* The first outcome measure, high burnout risk, was assessed using the validated Burnout Assessment Tool 12 (BAT-12) (23). BAT-12 comprises 12 items answered using a five-point Likert scale ranging from 1–5: “never” to “always”. The 12 items were compiled using a grand mean score, creating a total BAT-index (α:0.847). The BAT index was dichotomized using a validated cut-off value for a high risk of burnout >2.96, according to recommendations made by Schaufeli et al (24) who argue that individuals scoring above the cut-off value of BAT-12 most certainly will be diagnosed with clinical burnout if medically assessed.

Job dissatisfaction and turnover intention were measured using single items. Job dissatisfaction concerns the individual’s overall dissatisfaction with their job. Turnover intention concerned how often in the last 12 months the individual has considered applying for a new job. Each question was answered using a 5-point Likert scale. Job dissatisfaction was dichotomized as 1 (“rather dissatisfied” and “very dissatisfied”) or 0 (“very satisfied”, “rather satisfied” and “neutral”). Turnover intention was dichotomized as 1 (“every day”, “a few times a week” and “a few times a month”) or 0 (“sometimes the last 12 months” and “never”).

All three outcomes were assessed from the follow-up data in 2022.

*Potential confounders.* All potential confounders were assessed from baseline data in 2021 and applied with regard to a theoretical standpoint in WLI along with the potential confounders’ relation to mental health and perceived work environment.

Potential demographic confounders were sex and family situation (3, 25). Potential confounders concerning work environment included rank (14), working hours per week (3), and working overtime (26). Also, since our survey was collected during the pandemic, which had a significant impact on psychological distress among healthcare workers (6, 27), we also included work with COVID-19 patients as a potential confounder.

Age was omitted as a potential confounder due to its high correlation with rank (Pearson correlation 0.638) but included as a descriptive variable for demographic description.

Categories of the potential confounders can be found in table 1. Reference categories of each confounder for the logistics regression can be found in supplementary tables S2–4.

**Table 1:** Descriptive characteristics of the sample in baseline 2021 (N=1575).

Demographics		Total
		N (%)
Sex		
	Women	882 (56)
	Men	689 (44)
	Missing	0 (0)
Age (years)		
	27–37	410 (26.0)
	28–44	386 (24.5)
	45–55	391 (24.8)
	56–75	388 (24.6)
	Missing	0 (0)
Family situation		
	Living alone	158 (10.0)
	Living with only children	70 (4.4)
	Living with only partner	388 (24.6)
	Living with partner and children	943 (60.0)
	Missing	16 (1.0)
Rank		
	Physicians in training	477 (30.3)
	Specialist	641 (40.7)
	Consultants	451 (28.6)
	Missing	6 (0.4)
Experience (years)		
	<10	383 (24.3)
	10–15	327 (20.8)
	>15	727 (46.2)
	Missing	3 (0.2)
Working hours per week		
	<36	271 (17.2)
	34–45	837 (53.1)
	>45	465 (29.6)
	Missing	2 (0.1)
Working overtime		
	Rarely	293 (18.6)
	Several times/month	301 (19.1)
	Several times/week	972 (61.7)
	Missing	8 (0.5)
Work with COVID-19 patients		
	No	351 (22.3)
	Some shifts	350 (22.2)
	Entire pandemic	867 (55.0)
	Missing	6 (0.4)

### Statistical analysis

All analyses were performed using SPSS version 28.0 (IBM Corp, Armonk, NY, USA).

A missing value analysis was performed for all variables utilized in the analysis (supplementary table S4). Missing values percentage ranged from 0.0–2.7%. High risk of burnout in 2021 and 2022 were the only two variables with missing values >1% (1.9% and 2.7%, respectively) and were further analyzed in relation to demographic characteristics showing small differences in missing values: 1–2 percentage units. Missing values for descriptive variables are presented in table 1.

Descriptive summary statistics were used to describe the characteristics of the sample. Frequencies were computed to investigate the prevalence of WLI, high risk of burnout, job dissatisfaction and turnover intention, respectively. In addition, Chi^2^ (P<0.05) and McNemar (P<0.05) tests were performed to examine associations in prevalence.

To explore whether WLI is associated with a high risk of burnout, job dissatisfaction and turnover intention, we performed logistic regression analyses for each outcome. Crude models only tested for the specific variable towards each outcome. Model 1 included the exposure of WLI in 2021 as a continuous variable, adjusted for baseline high risk of burnout (continuous), job dissatisfaction (dichotomized) and turnover intention (dichotomized), respectively along with adjustments for potential confounders. Results from the regressions were presented as odds ratios (OR) with 95% confidence intervals (CI) and P-values (P<0.05).

## Results

### Demographics

Table 1 describes the characteristics of the analytical sample at baseline. More than half of the study participants were women (56%). Participants were aged 27–75 years, with a mean age of 47 (SD 11.6) years. The majority (60.5%) lived with a partner and children. It was most common to work as a specialist (40.1%). Nearly half of the physicians had >15 years of work experience.

Most physicians worked 34–45 hours per week and over 60% stated that they worked overtime several times per week. More than half of the physicians (55.3%) worked with COVID-19 patients.

### Prevalence of high risk of burnout, job dissatisfaction, turnover intention and WLI in 2021 and 2022

In table 2, the prevalence of WLI, high risk of burnout, job dissatisfaction and turnover intention is presented for each year, including significance levels *between* the categories for each variable in 2021 and significance levels for the change in prevalence between 2021 and 2022.

**Table 2:** Prevalence of work-life interference, high risk of burnout, job dissatisfaction and turnover intention, including significance levels between group categories and between years. **Text in bold indicates P-value <0.05**. [Pre=prevalence; OT=overtime.]

		Work-life interference					High risk of burnout					Job dissatisfaction					Turnover intention			
		Prev 2021 (%)	P-value ^a^	Prev 2022 (%)	P-value ^b^		Prev 2021 (%)	P-value ^a^	Prev 2022 (%)	P-value ^b^		Prev 2021 (%)	P-value ^a^	Prev 2022 (%)	P-value ^b^		Prev 2021 (%)	P-value ^a^	Prev 2022 (%)	P-value ^b^
Total		30.9	N/A	34.2	**0.003**		4.5	N/A	5.8	**0.038**		11.1	N/A	12.2	0.266		30.1	N/A	32.2	0.056
Sex																				
	Men	26.4	<0.001	30.2	**0.020**		4.1	0.579	5.2	0.345		10.0	0.210	11.4	0.337		28.1	0.136	31.3	0.055
	Women	34.5		37.3	0.071		4.7		6.3	0.082		12.0		12.7	0.582		31.6		32.9	0.401
Age (years)																				
	27–37	32.2	<0.001	36.7	**0.034**		5.2	**0.023**	7.0	0.248		11.5	0.156	13.7	0.306		33.9	**<0.001**	38.5	0.057
	38–44	28.6		34.1	**0.026**		4.0		5.0	0.523		10.9		13.5	0.221		33.1		38.3	**0.044**
	45–55	39.3		41.6	0.581		6.5		8,50	0.230		13.6		12.3	0.590		34.3		34.9	0.832
	56–75	23.4		24.3	0.603		2.1		2.7	0.754		8.5		9.1	0.742		18.8		16.8	0.312
Rank																				
	Consultant	30.7	0.574	32.6	0.740		3.4	0.225	3.4	0.118		9.3	0.165	8.0	0.337		25.9	**0.010**	25.6	0.820
	Specialist	29.8		32.8	0.275		4.3		6.5	0.054		11.0		13.4	0.241		29.4		33.4	0.202
	Physicians in training	32.8		38.6	**0.001**		5.7		7.8	0.804		13.2		15.2	0.219		25.9		25.6	0.055
Family situation																				
	Living alone	34.4	0.099	39.3	0.100		5.9	**0.019**	7.0	0.508		12.7	0.128	10.1	0.664		30.8	0.065	33.1	**0.049**
	Living with only children	37.7		34.3	1.00		11.6		10.4	1.00		18.6		17.6	1.00		31.4		33.8	0.727
	Living with only partner	26.4		30.1	0.328		3.9		4.0	1.00		9.3		11.5	0.635		24.7		25.1	1.00
	Living with partner and children	31.7		35.1	**0.016**		3.9		5.8	**0.018**		11.0		12.2	0.338		32.1		35.0	0.121
Time at work																				
	<36 work hours	18.5	<0.001	18.8	0.280		5.7	0.092	4.7	0.424		11.5	0.209	10.8	0.874		28.5	**<0.001**	29.5	0.071
	36–45 work hours	26.0		29.6	**0.045**		3.4		5.9	0.067		9.9		9.8	1.00		23.3		28.1	0.340
	>45 work hours	47.0		50.0	0.115		5.7		7.7	0.700		13.1		16.7	0.073		36.6		38.9	0.417
	OT - rarely	12.0	<0.001	15.5	0.511		3.2	0.271	2.4	0.073		3.1	**<0.001**	3.6	0.503		14.8	**<0.001**	16.3	1.00
	OT - sometimes	22.8		20.2	0.233		3.7		2.9	0.388		8.3		6.8	0.728		18.9		23.1	**0.009**
	OT - often	39.4		46.1	**0.018**		5.1		8.1	1.00		14.6		17.2	0.570		38.2		41.2	0.134
Work with COVID patients																				
	No	26.4	0.101	26.0	0.532		3.8	0.728	5.0	0.145		7.4	**0.041**	10.8	0.082		20.7	**<0.001**	21.0	**0.015**
	Some work shifts	31.5		33.5	0.328		5.0		6.5	0.359		12.3		11.5	0.874		34.0		34.4	0.912
	Entire pandemic	32.6		47.6	**0.006**		4.6		5.9	0.481		12.2		13.0	0.533		32.2		36.1	1.00

^a^ Between group-categories of 2021. ^b^ Between years (2021 vs. 2022) for each category.

Regarding prevalence found in table 2, the experience of WLI increased by 3.1 percentage points (P=0.003) while high risk of burnout increased by 1.3 percentage units (P=0.038) from 2021 to 2022. Although overall job dissatisfaction and turnover intention increased from 2021 to 2022 none were significant (P>0.05).

### Associations between WLI and subsequent high burnout risk, job dissatisfaction and turnover intention

Table 3 presents the results for the logistics regression testing the association between WLI and subsequent high burnout risk, job dissatisfaction and turnover intention. For full tables including values for confounders for each specific outcome, see supplementary (tables S2–4).

**Table 3:** Associations between experiences of work-life interference in 2021 (per one unit increase) and high risk of burnout, job satisfaction and Turnover intention in 2022, adjusted for potential confounders ^a^ **Text in italics indicates P-value <0.05**. [OR=odds ratio; CI=confidence interval.]

		Crude (unadjusted OR)			Model 1 ^a^			
		OR (95% CI)	P-value		OR (95% CI)	P-value	N	Nagelkerke R Square
High risk of burnout in 2022							1463	0.445
	Work-life interference 21 ^b^	3.67 (2.78–4.83)	**<0.001**		1.53 (1.05–2.25)	**0.029**		
	High risk of burnout 21 ^b^	19.98 (12.33–32.37)	**<0.001**		14.53 (8.39–25.16)	**<0.001**		
Job dissatisfaction in 2022							1524	0.269
	Work-life interference 21 ^b^	2.51 (2.10–2.99)	**<0.001**		2.06 (1.68–2.54)	**<0.001**		
	High job dissatisfaction 21 ^c^	9.51 (6.67–13.58)	**0.001**		5.82 (3.93–8.64)	**<0.001**		
Turnover intention in 2022							1525	0.478
	Work-life interference 21 ^b^	2.28 (2.02–2.58)	**<0.001**		1.72 (1.47–2.00)	**<0.001**		
	Turnover intention last day/week/month 21 ^c^	10.45 (8.14–13.42)	**<0.001**		7.37 (5.61–9.67)	**<0.001**		

^a^ Adjusted for sex, rank, family situation, time at work (work hours and overtime) and work with COVID-19 patients. ^b^ Continuous, scale 1–5. ^c^ Dichotomized. Reference: Low dissatisfaction/No turnover intention.

The results of model 1 (table 3), adjusted for baseline outcome and potential confounders, displayed that higher levels of WLI in 2021 were associated with 1.53 times higher odds of reporting a high risk of burnout (OR 1.53, 95% CI 1.05–2.25), 2.06 times higher odds of reporting job dissatisfaction (OR 2.06, 95% CI 1.68–2.54), and 1.72 times higher odds of reporting turnover intention in 2022 (OR 1.72, 95% CI 1.47–2.00).

## Discussion

This paper investigated the association between WLI, high risk of burnout and discontent with work (turnover intention and job dissatisfaction) among physicians in Sweden (2021–2022). This one-year follow-up data reveals that experiencing WLI in 2021 increases the likelihood of reporting a high risk of burnout, job dissatisfaction, and turnover intention in the subsequent year. Thus, our study indicates that WLI may be closely associated with a heightened risk of burnout and work discontent amongst Swedish physicians.

Our findings align with existing literature, which shows that experiences of WLI have been associated with a negative impact on mental health, including burnout and increased work discontent among physicians in other countries (3, 4, 12, 13, 16, 17, 28, 29). Research suggests that reversed causation (30) or reciprocal effects (31, 32) between WLI and health may also exist. It has also been suggested that individuals may become resilient to WLI over time (33). Meanwhile, research providing evidence that WLI is associated with subsequent medically certified sickness absence (18) indicates a negative health spiral. Nevertheless, this negative spiral and any cross-lagged effects should be further explored in our cohort with more data points. Moreover, the direction of effects between WLI and work discontent needs further research.

Data were collected during the COVID-19 pandemic, which had a large impact on the workload for some physicians and less on others. For example, due to the necessary focus on COVID-19-related tasks, physicians in surgical specialties were not able to carry out their work as usual (34, 35). Thus, physicians may have been affected by the pandemic regardless of whether they were working with COVID-19 patients or not (34). Therefore, to account for variations in workload and experiences, in addition to adjusting for work hours and overtime hours, we also adjusted for working with COVID-19 patients.

Both a high risk of burnout and work discontent hold critical consequences for the individual and the healthcare services. A high risk of burnout poses a direct threat to an individual’s health (36). At the societal level, the experience of having a high risk of burnout and work discontent following WLI may lead to an increased risk of sick leave (18, 19, 36) along with a potential reduction in the quality of care and risks to patient safety (7, 36, 37). These consequences come with a significant loss of competencies and economic costs for society (38). The mounting pressure on healthcare professionals, combined with a global shortage of healthcare workers (7), exacerbates the situation. Balancing work and private life is an ongoing struggle for many workers (17), yet for many, this balance does not affect the lives and health of others. Thus, the improvement of WLI is not just about safeguarding the health and competence of medical staff but in long-term also about the health and wellbeing of the patients.

### Strength and limitations

The longitudinal design of this article is also its major strength. This approach facilitated the adjustments for baseline burnout, job satisfaction and turnover intention in regression analysis, mitigating bidirectionality concerns. While the study benefits from two closely timed data points, it necessitates further investigation for a more extended perspective on the causal relation between WLI and a high risk of burnout as well as work discontent.

A potential weakness could lie in the use of single-item measures for job dissatisfaction and turnover intention, which has been criticized for oversimplifying complex human psychological constructs (39). However, validation studies by Fakunmoju (40) and Dolbier et al (41) found no difference in predictive validity between single items and multiple items when measuring job-related outcomes, especially when examining relatively stable attitudinal variables including the level of satisfaction and turnover intention (40).

Additionally, attrition bias needs to be addressed with regards to the attrition between surveys, essentially if psychosocial and clinical features of the physicians who only completed the baseline survey differ from those answering both baseline and follow. When analyzing the attrition between experiencing WLI and high risk of burnout at baseline with physicians who answered both time points, the mean values were similar, indicating low attrition bias.

Finally, unlike its predecessor such as the Maslach Burnout Inventory (MBI), the BAT offers a validated global burnout score and cut-off values, enhancing comparability across studies (1, 42). The distinction is important as longitudinal studies still need to be comparable between measures, yet many existing studies utilize the MBI (3, 25) or similar measures (4, 12), leading to discrepancies in reported burnout prevalence (1, 23). Thus, our use of the BAT contributes to longitudinal utility facilitating comparisons across different contexts and studies.

### Concluding remarks

This study found that experiences of WLI among Swedish physicians are associated with a subsequent high risk of burnout and discontent with work the following year.

To create healthy and sustainable healthcare, all physicians need to be able to balance their careers with their private lives. Considering our findings and the consequences of experiencing both a heightened risk of burnout and work discontent, such as negatively affected quality of care and patient safety, additional studies within the Swedish context are needed to study further the role of WLI among healthcare workers and its healthcare outcomes.

### Ethics approval and consent to participate

The Swedish Ethical Review Authority approved the LOHHCS study (2020-06613; 2021-05574-02). All physicians gave their consent when they logged in to the web survey or posted the paper survey.

## Supplementary material

Supplementary material

## Data Availability

Due to ethical reasons, the datasets analyzed during the current study are not publicly available but are available from the corresponding author upon reasonable request.
