# Supplementary material for: Impact of work–life interference on burnout and job discontent: A one-year follow-up study of physicians in Sweden
Source: Scand J Work Environ Health. 2024 Sep 30;50(7):519–26. doi: 10.5271/sjweh.4181 (PMC11473125; doi:10.5271/sjweh.4181)
Supplement: Supplementary material [file SJWEH-50-519-S001.pdf]

Impact of work–life interference on burnout and job discontent: A one-year follow-up study of physicians in Sweden[1]

by Britta E Gynning, MS,[2] Filip Christiansen, MD, Ulrik Lidwall PhD, Emma Brulin, PhD

1. Supplementary material
2. Correspondence to: Britta E Gynning, IMM, Karolinska Institutet, Nobels väg 13, 171 65 Solna, Sweden. [E-mail: Britta.elsert.gynning@ki.se]

Table S1. Exposure and outcome items

EXPOSURE	ITEM	RESPONSE ALTERNATIVES
Work Life Interference	When I come home from work, I am too tired to do the things I would like to do.	1=Not at all; 2=Rarely; 3=Sometimes; 4=Often; 5=Almost all the time
	My private life does not look the way I would like it to because of my work.	1=Not at all; 2=Rarely; 3=Sometimes; 4=Often; 5=Almost all the time
	I overlook personal problems because of the demands of my work.	1=Not at all; 2=Rarely; 3=Sometimes; 4=Often; 5=Almost all the time
	My personal life suffers because of my work.	1=Not at all; 2=Rarely; 3=Sometimes; 4=Often; 5=Almost all the time
	I change and adapt my personal life to the demands of the workplace.	1=Not at all; 2=Rarely; 3=Sometimes; 4=Often; 5=Almost all the time
OUTCOME	ITEM	RESPONSE ALTERNATIVES
BAT-12	I feel mentally exhausted	1=Not at all; 2=Rarely; 3=Sometimes; 4=Often; 5=Almost all the time
	I find it hard to recover my energy	1=Not at all; 2=Rarely; 3=Sometimes; 4=Often; 5=Almost all the time
	I feel physically exhausted	1=Not at all; 2=Rarely; 3=Sometimes; 4=Often; 5=Almost all the time
	I struggle to find any enthusiasm for my work	1=Not at all; 2=Rarely; 3=Sometimes; 4=Often; 5=Almost all the time
	I feel a strong aversion towards my job	1=Not at all; 2=Rarely; 3=Sometimes; 4=Often; 5=Almost all the time
	I'm cynical about what my work means to others	1=Not at all; 2=Rarely; 3=Sometimes; 4=Often; 5=Almost all the time
	I have trouble staying focused	1=Not at all; 2=Rarely; 3=Sometimes; 4=Often; 5=Almost all the time
	I have trouble concentrating	1=Not at all; 2=Rarely; 3=Sometimes; 4=Often; 5=Almost all the time
	I make mistakes because I have my mind on other things	1=Not at all; 2=Rarely; 3=Sometimes; 4=Often; 5=Almost all the time
	I feel unable to control my emotions	1=Not at all; 2=Rarely; 3=Sometimes; 4=Often; 5=Almost all the time
	I do not recognize myself in the way I react emotionally	1=Not at all; 2=Rarely; 3=Sometimes; 4=Often; 5=Almost all the time
	I may overreact unintentionally	1=Not at all; 2=Rarely; 3=Sometimes; 4=Often; 5=Almost all the time
Job Dissatisfaction	How satisfied or dissatisfied are you with your job?	1=Very satisfied; 2=Quite satisfied; 3=Neither satisfied nor dissatisfied; 4=Quite dissatisfied; 5=Very dissatisfied
Turnover Intention	In the last 12 months, how often have you considered looking for a new job?	1=Every day; 2=Some/few times a week; 3= Some/few times a month; 4= Some/few times in the last 12 months; 5=Never

Table S2: Associations between experiences of Work-Life Interference in 2021 (per one unit increase) and High risk of burnout in 2022, adjusted for potential confounders*

	**Crude (Unadjusted OR)**		**Model 1***	
**n**			1463	
	OR(95% CI)	P-value	OR(95% CI)	P-value
**Work-Life Interference 21‡**	*3.67(2.78-4.83)*	*<.001*	*1.53(1.05-2.25)*	*0.029*
**High risk of burnout 2021‡**	*19.98(12.33-32.37)*	*<0.001*	*14.53(8.39-25.16)*	*<0.001*
**Sex**				
Women (ref. Men)	1.23(0.79-1.92)	0.355	0.96(0.54-1.69)	0.880
**Rank**				
Consultant (ref.)	*1*	*0.008*	*1*	*0.042*
Specialists	*1.91(1.02-3.58)*	*0.043*	2.19(0.97-4.96)	0.061
Physicians in training	*2.79(1.44-5.05)*	*0.002*	*2.84(1.26-6.42)*	*0.012*
**Family situation**				
Living alone (ref.)	1		1	0.783
Living with only children	1.04(0.38-2.85)	0.947	0.77(0.18-3.21)	0.718
Living with only a partner	*0.45(0.21-0.96)*	*0.039*	0.59(0.21-1.64)	0.309
Living with a partner and children	0.67(0.35-1.25)	0.207	0.77(0.33-1.83)	0.560
**Time at work**				
Work hours <36h (ref.)	1	0.246	1	0.410
Work hours 36-45h	0.66(0.38-1.17)	0.154	0.61(0.29-1.29)	0.197
Work hours >45h	0.93(0.51-1.68)	0.799	0.61(0.26-1.44)	0.260
Overtime - rarely (ref.)	1	0.560	1	0.560
Overtime - sometimes	1.51(0.64-3.55)	0.343	1.70(0.55-5.23)	0.357
Overtime - often	*2.22(1.09-4.52)*	*0.028*	1.18(0.44-3.14)	0.739
**Work with COVID-19 patient**				
No (ref.)	1	0.698	1	0.787
Some work shifts	1.32(0.69-2.54)	0.400	0.94(0.41-2.18)	0.893
Entire pandemic	1.20(0.68-2.11)	0.533	0.80(0.38-1.67)	0.548
**Nagelkerke R Square**			0.445	

Text in italics indicated p-value <0.05

‡ Continuous, scale 1-5

*Adjusted for sex, rank, family situation, time at work (work hours & overtime) & work with COVID-19 patients

Table S3: Associations between experiences of Work-Life Interference in 2021 (per one unit increase) and Job Dissatisfaction in 2022, adjusted for potential confounders*				
	**Crude (Unadjusted OR)**		**Model 1***	
**n**			1524	
	OR(95% CI)	P-value	OR(95% CI)	P-value
**Work-Life Interference 21‡**	*2.51(2.10-2.99)*	*<0.001*	*2.06(1.68-2.54)*	*<0.001*
**High Job Dissatisfaction 21†**	*9.51(6.67-13.58)*	*0.001*	*5.82(3.93-8.64)*	*<0.001*
**Sex**				
Women (ref. Men)	1.13(0.83-1.53)	0.441	0.87(0.61-1.24)	0.435
**Rank**				
Consultant (ref.)	*1*	*0.001*	*1*	*0.004*
Specialists	*1.71(1.13-2.61)*	*0.011*	*1.90(1.18-3.08)*	*0.009*
Physicians in training	*2.22(1.45-3.39)*	*<.001*	*2.27(1.38-3.74)*	*0.001*
**Family situation**				
Living alone (ref.)	1	0.254	1	0.925
Living with only children	1.21(.56-2.59)	0.631	0.98(0.39-2.47)	0.974
Living with only a partner	0.65(0.38-1.13)	0.130	0.82(0.44-1.53)	0.528
Living with a partner and children	0.39(0.50-1.31)	0.393	0.88(0.51-1.53)	0.652
**Time at work**				
Work hours <36h (ref.)	*1*	*0.005*	*1*	*0.051*
Work hours 36-45h	0.79(0.52-1.22)	0.286	*0.55(0.34-0.91)*	*0.019*
Work hours >45h	1.39(.89-2.15)	0.147	0.72(0.42-1.24)	0.239
Overtime - rarely (ref.)	*1*	*<0.001*	1	0.213
Overtime – sometimes	*2.20(1.12-4.34)*	*0.023*	1.55(0.75-3.21)	0.241
Overtime – often	*3.86(2.15-6.91)*	*<0.001*	1.77(0.93-3.37)	*0.081*
**Work with COVID-19 patient**				
No (ref.)	1	0.499	1	0.441
Some work shifts	1.07(0.67-1.72)	0.280	0.70(0.41-1.22)	0.210
Entire pandemic	1.24(0.84-1.83)	0.769	0.79(0.50-1.26)	0.322
**Nagelkerke R Square**			0.269	

Text in italics indicates p-value <0.05

‡ Continuous, scale 1-5

† Dichotomized, Ref. Low dissatisfaction

*Adjusted for sex, rank, family situation, time at work (work hours & overtime) & work with COVID-19 patients

Table S4: Associations between experiences of Work-Life Interference in 2021 (per one unit increase) and Turnover Intention in 2022, adjusted for potential confounders*				
	**Crude (Unadjusted OR)**		**Model 1***	
**n**			1525	
	OR(95% CI)	P value	OR(95% CI)	P value
**Work-Life Interference 21**‡	*2.28(2.02-2.58)*	*<0.001*	*1.72(1.47-2.00)*	*<0.001*
**Turnover Intention last day/week/month 21**†	*10.45(8.14-13.42)*	*<0.001*	*7.37(5.61-9.67)*	*<0.001*
**Sex**				
Women (ref. Men)	1.08(0.87-1.34)	0.485	0.86(0.66-1.12)	0.250
**Rank**				
Consultant (ref.)	*1*	*<0.001*	*1*	*0.011*
Specialists	*1.39(1.06-1.82)*	*0.018*	1.35(0.96-1.89)	0.085
Physicians in training	*1.95(1.47-2.58)*	*<0.001*	*1.72(1.2-2.45)*	*0.003*
**Family situation**				
Living alone (ref.)	*1*	*0.002*	1	0.048
Living with only children	0.640(.35-1.17)	0.146	0.52(0.24-1.11)	0.091
Living with only a partner	*0.52(0.35-0.78)*	*<0.001*	0.53(0.33-0.85)	0.009
Living with a partner and children	0.84(0.59-1.18)	0.313	0.71(0.46-1.08)	0.11
**Time at work**				
Work hours <36h (ref.)	*1*	*0.002*	1	0.205
Work hours 36-45h	1.07(0.79-1.45)	0.651	0.71(0.49-1.04)	0.075
Work hours >45h	*1.58(1.14-2.18)*	*0.006*	0.76(0.5-1.17)	0.218
Overtime - rarely (ref.)	*1*	*<0.001*	1	0.746
Overtime – sometimes	*1.55(1.05-2.30)*	*<0.001*	1.18(0.75-1.86)	0.475
Overtime – often	*2.70(1.96-3.73)*	0.027	1.15(0.77-1.71)	0.493
**Work with COVID-19 patient**				
No (ref.)	*1*	*<0.001*	*1*	*0.019*
Some work shifts	*1.97(1.40-2.76)*	*<0.001*	*1.56(1.04-2.36)*	*0.033*
Entire pandemic	*2.12(1.58-2.84)*	*<0.001*	*1.67(1.16-2.39)*	*0.005*
**Nagelkerke R Square**			0.478	

Text in italics indicates p-value <0.05

‡ Continuous, scale 1-5

† Dichotomized, Ref. Low dissatisfaction

*Adjusted for sex, rank, family situation, time at work (work hours & overtime) & work with COVID-19 patients

Table S4. Missing Value Analysis of Work-Life Interference, High Risk of Burnout, Job Dissatisfaction and Turnover Intention								
	Work-Life Interference		High Risk of Burnout 21		Job Dissatisfaction		Turnover Intention	
	2021	*2022*	*2021*	*2022*	*2021*	*2022*	*2021*	*2022*
	%	*%*	*%*	*%*	*%*	*%*	*%*	*%*
Missing Values	10(0.6)	14(0.9)	30(1.9)	43(2.7)	4(0.3)	3(0.2)	5(0.3)	3(0.2)
**Sex**								
Men	0.9	1.0	2.2	2.5	0.4	0.1	0.3	0.1
Women	0.5	0.8	1.7	2.9	0.1	0.2	0.3	0.2
**Age**								
27-37	0.7	0.2	1.2	2.4	0.2	0.0	0.7	0.0
28-44	0.3	0.5	1.8	1.8	0.3	0.3	0.5	0.5
45-55	0.5	1.5	2.3	3.3	0.5	0.0	0.0	0.3
56-75	1.0	1.3	2.3	3.4	0.0	0.5	0.0	0.0
**Family Situation**								
Living alone	0.6	1.9	3.2	3.8	0.0	0.6	1.3	0.0
Living with only children	1.4	1.4	1.4	2.9	0.0	0.0	0.0	0.0
Living with only partner	0.5	0.5	2.1	3.4	0.0	0.3	0.0	0.0
Living with partner and children	0.6	0.7	1.6	2.3	0.4	0.1	0.3	0.3
**Rank**								
Physicians in training	0.8	0.6	1.3	2.5	0.2	0.2	0.6	0.2
Specialist	0.2	0.9	2.3	2.8	0.3	0.2	0.3	0.2
Consultants	1.1	0.9	2.0	2.9	0.2	0.2	0.0	0.2
**Workhours**								
Under 36h/w	0.0	0.7	3.0	2.6	0.4	0.4	0.4	0.0
34-45h/w	1.1	0.6	1.6	2.3	0.2	0.1	0.5	0.4
Over 45h/w	0.2	1.5	1.9	3.7	0.2	0.2	0.0	0.0
**Overtime per week**								
Rarely	0.4	1.0	2.1	2.4	0.4	0.1	0.2	0.1
Several times/m	1.3	0.7	0.7	2.6	0.0	0.3	0.0	0.0
Several times/w	0.3	0.3	2.7	4.1	0.0	0.3	0.7	0.7
**Work with COVID-19 patients**								
No	0.6	0.8	1.7	2.4	0.3	0.1	0.5	0.2
Some shifts	0.3	1.1	2.3	3.1	0.3	0.6	0.0	0.3
Entire pandemic	0.9	0.9	1.7	3.1	0.0	0.0	0.0	0.0
